# Validation of Artificial Intelligence–enhanced Stimulated Raman Histopathology for Intraoperative Margin Assessment During Robot-assisted Radical Prostatectomy: Preliminary Results from the ROBOSPEC Study

**DOI:** 10.1016/j.euros.2025.10.022

**Published:** 2025-11-13

**Authors:** Arif Özkan, Karl-Moritz Schröder, Peter Bronsert, Julia Franz, Maximilian Glienke, August Sigle, Jürgen Beck, Martin Werner, Christian Gratzke, Jakob Straehle, Samir S. Taneja, Miles P. Mannas, Nikolaos Liakos

**Affiliations:** a Department of Urology, University of Freiburg Medical Center, Freiburg, Germany; b Faculty of Medicine, University of Freiburg, Freiburg, Germany; c Department of Urology, Koç University Hospital, Istanbul, Turkey; d Institute for Surgical Pathology, University of Freiburg Medical Center, Freiburg, Germany; e Histopathology and Digital Pathology Core Facility, University of Freiburg Medical Center, Freiburg, Germany; f Tumorbank Comprehensive Cancer Center, University of Freiburg Medical Center, Freiburg, Germany; g Department of Neurosurgery, University of Freiburg Medical Center, Freiburg, Germany; h Center for Advanced Surgical Tissue Analysis, University of Freiburg Medical Center, Freiburg, Germany; i Department of Urology, NYU Langone Health, New York, NY, USA; j Department of Radiology, NYU Langone Health, New York, NY, USA; k Department of Biomedical Engineering, NYU Langone Health, New York, NY, USA; l Department of Urologic Sciences, University of British Columbia, Vancouver, Canada; m Mohseni Institute of Urologic Sciences, Vancouver Prostate Centre, University of British Columbia, Vancouver, Canada

**Keywords:** Robot-assisted prostatectomy, Intraoperative frozen sections, Stimulated Raman spectroscopy, Artificial intelligence

## Abstract

**Background and objective:**

Stimulated Raman histology (SRH) offers promising near–real-time tissue visualization for intraoperative pathology assessment. We present preliminary results from the ROBOSPEC study, with a focus on the accuracy of results obtained via an integrated artificial intelligence (AI) tool.

**Methods:**

ROBOSPEC is a prospective, single-arm pilot study involving patients with prostate cancer undergoing robot-assisted radical prostatectomy (RARP). Probes from the RP specimens from the first 18 patients with intermediate-risk or high-risk prostate cancer were collected bilaterally from the dorsolateral sides of the prostate and examined with frozen section with hematoxylin and eosin staining (cryo-HE), SRH imaging (NIO laser imaging system, Invenio Imaging, Santa Clara, CA, USA). A previously published New York University AI algorithm (NYU-AI) that is based on the Inception-ResNet-v2 CNN architecture was used to generate three-color overlays to assist in interpretation. SRH images were reviewed by blinded urologists using this AI-enhanced output.

**Key findings and limitations:**

NYU-AI identified positive surgical margins in 22% of patients, with no statistically significant difference in comparison to cryo-HE (*p* > 0.05). Patient-based analysis yielded sensitivity and a negative predictive value (NPV) of 1.0, specificity of 0.93, and a positive predictive value of 0.75. Sample-based analysis showed similar performance, with specificity of 0.97 and identical sensitivity and NPV. These findings indicate strong diagnostic agreement between NYU-AI and conventional intraoperative pathology. Limitations of the study include the small patient cohort, the single-center design, previous training of the NYU-AI tool on prostate biopsy and periprostatic surgical-bed samples, and the lack of testing of interobserver agreement.

**Conclusions and clinical implications:**

Our preliminary findings support the potential of SRH with NYU-AI for intraoperative detection of positive surgical margins during RARP. Implementation of this technique should be further discussed after more studies have been conducted.

**Patient summary:**

We looked at an artificial intelligence program using a method called stimulated Raman histology to assess the cancer status of the cutting margin during robot-assisted surgery to remove the prostate. Our preliminary results show that this method could be an alternative to the current standard as it provides accurate and faster results.

## Take Home Message

Our preliminary findings suggest that simulated Raman histology (SRH) with artificial intelligence assistance can support intraoperative decision-making and demonstrate potential for SRH feasibility in surgical margin assessment. While these results are encouraging, ongoing direct comparisons to frozen sections stained with hematoxylin and eosin and to paraffin-embedded sections are essential to further validate the findings and enhance the reliability of this strategy.

## 1 Introduction

Robot-assisted radical prostatectomy (RARP) is a standard treatment for localized prostate cancer [1,2]. One of the key intraoperative challenges in RARP is reliable and timely assessment of surgical margins, especially in patients at high risk of locally advanced disease [3]. Conventional assessment using hematoxylin and eosin staining of frozen sections is currently the standard for intraoperative evaluation of resection margins. The technique involves immediate freezing of the tissue to be examined and subsequent use of a microtome and staining for microscopic assessment [4]. However, this approach has some limitations, such as extensive time requirements (∼30 min per sample), potential for artifact occurrence during freezing, staining, and tissue processing, and a possible human error source due to assessor subjectivity during interpretation, which can lead to diagnostic errors and inadequate assessment of resection margins [5,6]. Stimulated Raman histology (SRH) is an emerging and promising strategy that provides an objectifiable and reproducible technique for intraoperative assessment of tissue [7]. SRH uses the intrinsic vibrations of molecules within the tissue under investigation to generate high-resolution imaging via resonance Raman scattering [8]. This process allows rapid and nondestructive visualization of cellular and subcellular structures and molecular composition without a need for staining or complex sample preparation [9]. SRH offers a near–real-time, label-free imaging technique that has shown promise in many surgical fields [9,10]. Previous studies have demonstrated the ability of SRH to distinguish benign from malignant prostate tissue, and AI-enhanced SRH has been applied for margin assessment in the surgical bed following RARP [[11], [12], [13], [14], [15]]. However, no study to date has validated whether the same AI model can accurately interpret SRH images derived directly from prostatectomy specimens.

The aim of the ROBOSPEC trial is to show the applicability of the AI-enhanced SRH to samples taken directly from prostatectomy specimens. In this study, our goal was to validate the AI model developed by Mannas et al [14] for use on prostate tissue excised during RARP using our initial results. Unlike the original model, which was trained and tested on SRH images from periprostatic surgical-bed samples and needle biopsies, we applied the AI-enhanced SRH method to assess margin samples obtained directly from the excised prostate gland. By evaluating its performance against conventional frozen-section and paraffin-embedded histology, this study tests the robustness and generalizability of the NYU-AI approach in a new anatomic context.

## 2 Patients and methods

This prospective, single-arm, single-center validation study was conducted at the University of Freiburg Medical Centre. Patients were enrolled consecutively between September 1, 2024, and October 31, 2024, as part of a prospective validation protocol. Inclusion criteria were confirmed written consent, histologically confirmed prostate adenocarcinoma classified as intermediate or high risk according to the European Association of Urology classifications, and RARP scheduled at our institution. Exclusion criteria were prior prostate surgery or pelvic radiotherapy, or the presence of rare histological subtypes. All patients provided written informed consent before enrollment.

After prostatectomy, the specimen was immediately sent to the pathology department for frozen section analysis. For standard intraoperative assessment (P1), bilateral tissue samples were taken from the dorsolateral prostate margins, anatomically corresponding to areas adjacent to the neurovascular bundles following frozen section. These samples were approximately 15–20 mm in length and 2–3 mm in thickness, oriented perpendicularly to the prostatic capsule. For the experimental evaluation (P2), SRH specimens were collected from adjacent areas located immediately medial to the P1 sampling line, along the same dorsolateral plane of the prostate. Care was taken to maintain consistent orientation, depth, and anatomic correlation between P1 and P2 to allow valid intrapatient comparisons. Both P1 and P2 samples were collected using a standardized protocol by the same pathologist in collaboration with the surgical team to minimize variability.

Nerve-sparing techniques were applied on the basis of preoperative risk stratification, imaging findings, and intraoperative judgment. Depending on oncological suitability, unilateral or bilateral nerve-sparing procedures were applied. The specific approach (intrafascial, interfascial, or high-release) was chosen according to surgeon preference and patient anatomy, but was not standardized across the cohort. These variables were not controlled for in the current analysis and may influence surgical margin status.

**Fig. 1:**
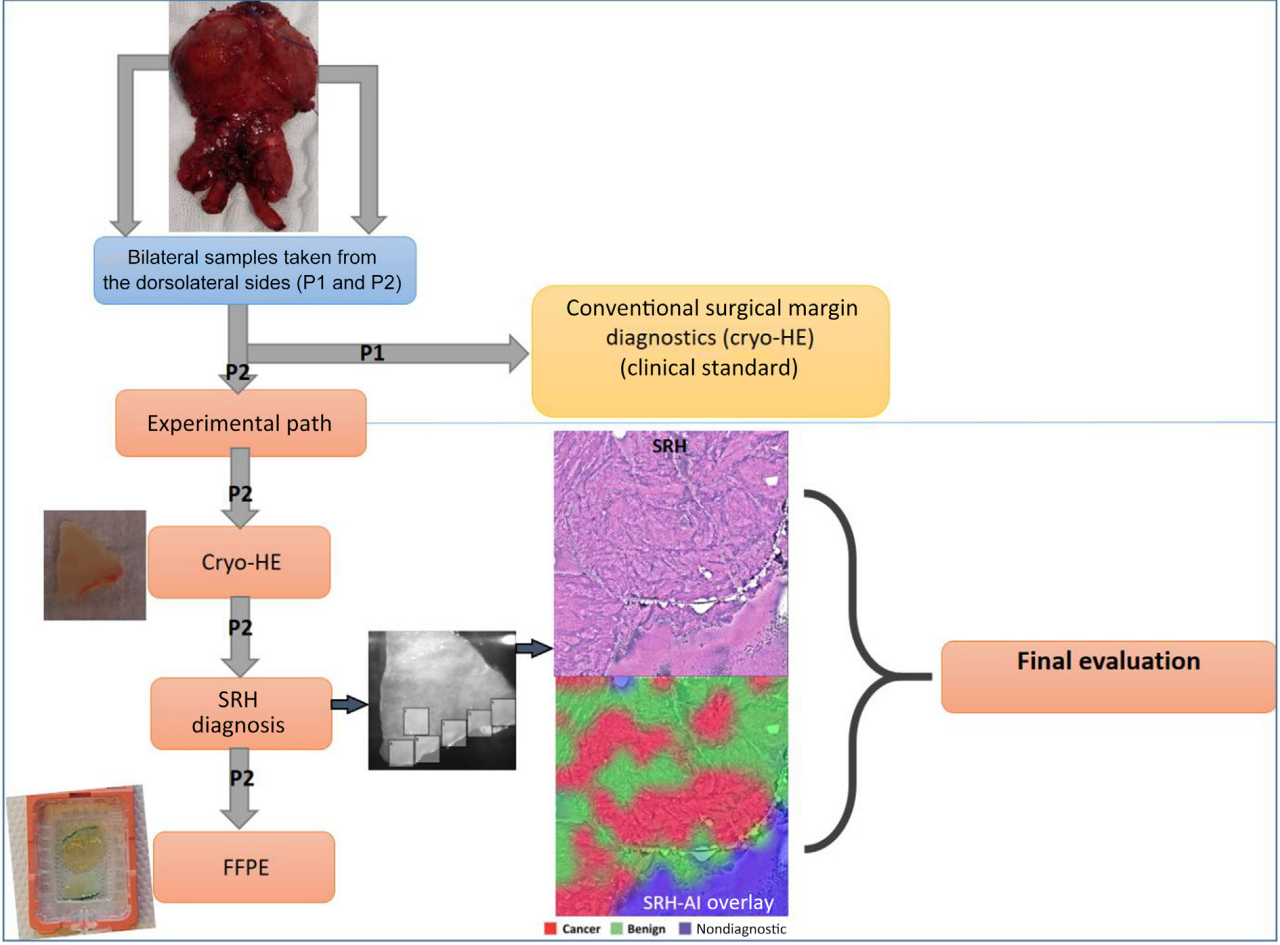
Workflow for surgical margin assessment using NYU-AI versus conventional cryo-HE pathology. After prostatectomy, dorsolateral margin samples were obtained bilaterally. Samples from region P1 underwent standard clinical analysis of frozen sections stained with hematoxylin and eosin (cryo-HE). Adjacent samples from region P2 were used in the experimental path and underwent sequential cryo-HE, stimulated Raman histology (SRH), and formalin-fixed, paraffin-embedded (FFPE) histopathology. SRH images were analyzed using an artificial intelligence AI-based diagnostic model with overlaid classification maps (cancer = red; benign = green; nondiagnostic = gray). NYU-AI = New York University artificial intelligence tool.

**Fig. 2:**
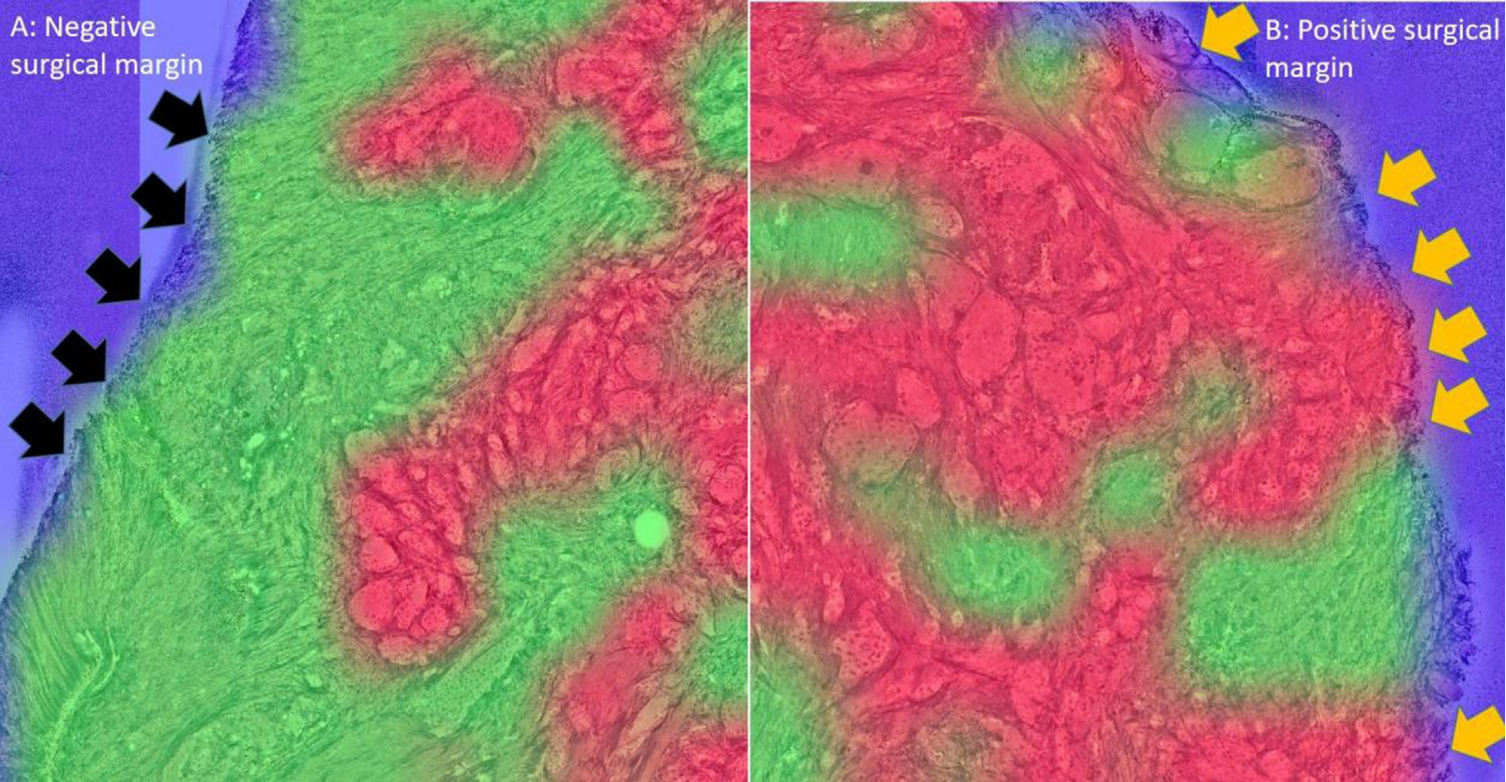
NYU-AI–based detection of surgical margins. (A) Negative surgical margin: no red-colored tumor regions are present at the outer surface of the specimen (black arrows). (B) Positive surgical margin: red-colored regions indicating tumor cells reach the specimen surface (orange arrows), fulfilling the NYU-AI criteria for a positive margin. NYU-AI = New York University artificial intelligence tool.

Each specimen was processed in three ways (Fig. 1): (1) frozen section with hematoxylin and eosin staining (cryo-HE); (2) SRH imaging using the CE-certified NIO laser imaging system (Invenio Imaging, Santa Clara, CA, USA); and (3) standard formalin-fixed, paraffin-embedded (FFPE) histopathology. SRH images were processed using the convolutional neural network–based New York University AI algorithm (NYU-AI) developed by Mannas et al [14], which generates three-color overlays to differentiate malignant, benign glandular, and stromal tissue components (Fig. 2) [[12], [13], [14], [15]].

A urologist blinded to the pathology results interpreted the AI-enhanced SRH images as previously described [14]. The margin status was classified as either positive or negative. We defined a positive surgical margin as the presence of tumor cells (indicated by red-colored patches according to NYU-AI) at the outer surface of the specimen (Fig. 2). For diagnostic comparison, NYU-AI outputs were evaluated against the binary margin status (positive vs negative) as determined by the attending pathologist for intraoperative cryo-HE sections. No pixel-wise or region-level annotation comparison was performed in this stage. The diagnostic performance of NYU-AI was evaluated by comparing its results with the gold-standard cryo-HE results. The sensitivity, specificity, positive predictive value (PPV), and negative predictive value (NPV) were calculated. To assess diagnostic agreement between NYU-AI and the reference method (cryo-HE), McNemar’s test was used for paired categorical data. The test was applied separately to both patient-based and sample-based comparisons of positive and negative surgical margin classifications. A *p* value <0.05 was considered statistically significant.

## 3 Results

A total of 18 patients were enrolled in the first phase of the study. Mean age was 58.1 yr and median PSA was 9.5 ng/ml (interquartile range 9.1; Table 1). A total of 36 margin samples were collected, of which three were excluded because of artifacts, resulting in 33 evaluable samples. From these, 141 SRH images were generated, with an average of 4.5 images acquired per sample. Of the 33 samples analyzed, 29 were imaged using the 1.7 mm × 1.8 mm setting, and four with the 2.5 mm × 2.8 mm setting. The average image generation time per sample was 8.2 min. Three samples were excluded because of artifacts; all were imaged at 1.7 mm × 1.8 mm (Table 1).

**Table 1:** Clinicopathological and imaging characteristics of the study cohort

Parameter	Result
Patients (*n*)	18
Mean age (yr)	58.1
Median prostate-specific antigen, ng/ml (interquartile range)	9.5 (9.1)
Margin samples collected (*n*)	36
Margin samples analyzed after exclusions (*n*)	33
SRH images generated (*n*)	141
SRH image size (*n*)	
1.7 mm × 1.8 mm	29
2.5 mm × 2.8 mm	4
Samples excluded because of artifacts (*n*)	3^a^
Mean number of SRH images per sample (*n*)	4.5
Mean time for NYU-AI SRH image generation (min)	8.2
Mean image coverage per margin (mm)	8.11
Positive surgical margins detected, *n* (%)	
NYU-AI	4 (22)
Cryo-HE	3 (17)
pT stage, *n* (%)	
pT2	11 (61)
pT3a	4 (22)
pT3b	3 (17)
Positive surgical margin cases on cryo-HE by pT stage, *n* (%)	
pT2	1 (33.3)
pT3a	1 (33.3)
pT3b	1 (33.3)

Cryo-HE = analysis of frozen sections stained with hematoxylin and eosin; NYU-AI = New York University artificial intelligence tool; SRH = stimulated Raman histopathology; AI = artificial intelligence.

a All 1.7 mm × 1.8 mm.

NYU-AI detected positive surgical margins (PSMs) in 4/18 patients (22%). One false-positive NYU-AI result was observed. Postoperative histopathological analysis showed that 11 patients (61%) had pT2, four (22%) had pT3a, and three (17%) had pT3b disease. Among the four patients with PSMs, one (25%) had pT2, one (25%) had pT3a, and two (50%) had pT3b cancer, indicating that PSMs were present even in organ-confined tumors (Table 1).

**Fig. 3:**
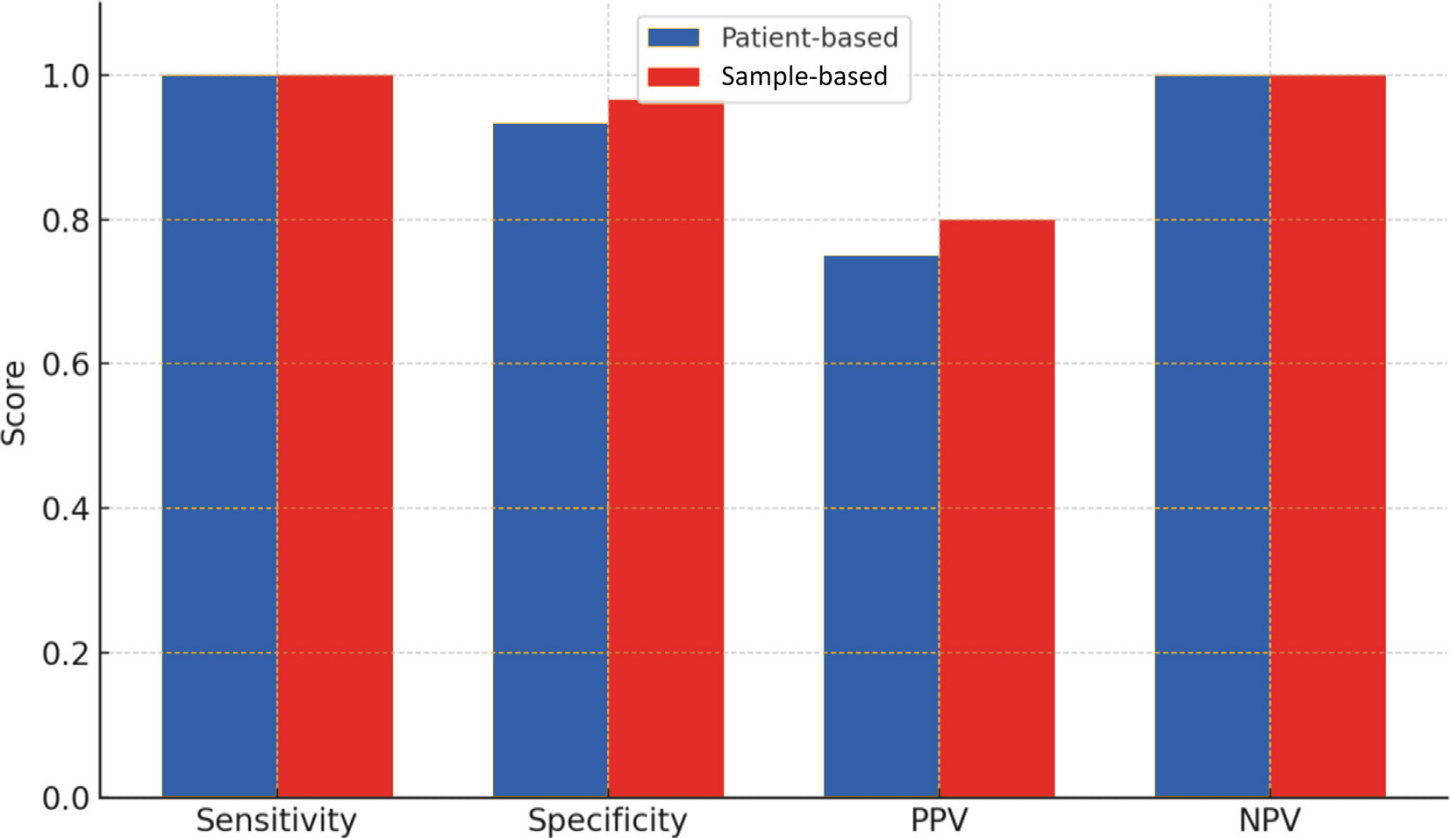
Diagnostic performance of NYU-AI in patient-based versus sample-based analysis. NPV = negative predictive value; NYU-AI = New York University artificial intelligence tool; PPV = positive predictive value.

Patient-based analysis demonstrated high diagnostic performance of NYU-AI, with sensitivity and NPV of 1.0, specificity of 0.93, and PPV of 0.75. Concordance with cryo-HE according to McNemar’s test was statistically significant (p > 0.05; Table 2 and Fig. 3).

**Table 2:** Diagnostic performance and concordance of NYU-AI in detecting positive surgical margins

Analysis type	Sensitivity	Specificity	PPV	NPV	McNemar *p* value
Patient-based	1.00	0.933	0.75	1.00	>0.05
Sample-based	1.00	0.966	0.75	1.00	>0.05

NPV = negative predictive value; NYU-AI = New York University artificial intelligence tool; PPV = positive predictive value.

Sample-based analysis yielded similarly promising results. NYU-AI demonstrated sensitivity and NPV of 1.0, specificity of 0.97, and PPV of 0.75. Statistical agreement with cryo-HE was highly significant (*p* > 0.05; Table 2 and Fig. 3).

## 4 Discussion

These initial results from the ongoing ROBOSPEC study validate the use of AI-enhanced SRH on prostatectomy specimens, which extends the application of the model originally developed by Mannas et al [14]. Our findings demonstrate that the same AI algorithm retains its diagnostic accuracy when applied to excised tissue margins and achieves excellent sensitivity and specificity. This supports the generalizability of the model and suggests that NYU-AI can be effectively implemented across different anatomic settings. SRH imaging was deliberately confined to well-defined dorsolateral regions adjacent to the standard surgical margin specimens, with average coverage of approximately 8.1 mm per sample. This approach allowed for anatomically matched comparisons between NYU-AI and cryo-HE in a controlled and standardized setting. In comparison to the cryo-HE reference standard, NYU-AI achieved high concordance, with sensitivity and NPV of 1.0, specificity of 0.93 (patient-based) and 0.97 (sample-based), and PPV of 0.75 in both analyses. Notably, sample-based evaluation revealed slightly higher specificity and NPV than patient-based analysis, which underscores the robustness of the NYU-AI performance. These results highlight the potential of NYU-AI for reliable intraoperative detection of PSMs across a range of pathological stages and support its utility as a complementary tool for real-time surgical margin assessment during RARP.

The implications of this validation are significant. First, analysis of excised specimen margins aligns more closely with traditional pathological workflows, which makes this a practical and feasible solution. This approach avoids challenges related to surgical bed imaging, such as limited access, bleeding, and field contamination, which can affect image quality. Moreover, NYU-AI integration into specimen-based intraoperative workflows could facilitate consistent and high-quality tissue assessment, with potential for standardized implementation across institutions. The high NPV of 1.0 we observed suggests that NYU-AI could serve as a reliable tool to confirm negative margins intraoperatively. This is crucial for nerve-sparing procedures, as avoiding unnecessary resection of neurovascular bundles can preserve erectile and urinary function without compromising oncologic outcomes [2,3]. Our results also emphasize that PSMs can occur even in pT2 tumors, which reinforces the relevance of intraoperative margin assessment regardless of tumor stage. Identification of these cases in real time could enable surgeons to perform immediate re-resection and could potentially reduce the need for adjuvant therapy.

Our study contributes to a growing body of literature that supports the feasibility of integration of AI-assisted digital pathology tools in the operating room [10,14,15]. The portable and rapid nature of SRH imaging, combined with trained AI models, means that surgeons could make faster, evidence-based decisions, even in settings without immediate pathologist availability. Nonetheless, one discordant case in our cohort revealed the need for cautious interpretation. Although NYU-AI highlighted small, focal areas directly on the surgical margin, no tumor was confirmed on cryo-HE, and the margin was classified as R0. This false-positive finding may have resulted from misclassification of benign intraprostatic glands as malignant, which is a known limitation of the current AI model. Occasionally, the algorithm flags benign glandular structures as suspicious, particularly in the absence of surrounding malignant morphology [12]. As the AI model provides only probability-based visual overlays without regional quantification, it remains unclear whether this discordance reflects an overcall by the algorithm or a user interpretation bias. Further experience and prospective validation will be essential to address such cases and reduce interpretive variability. Future directions include larger-scale validation in multi-institutional cohorts, AI training on more diverse data sets, and exploration of how NYU-AI integration affects long-term oncological outcomes, patient satisfaction, and health care costs.

Unlike frozen sections, which are time- and resource-intensive [5,6], SRH imaging with AI support allows near–real-time interpretation with minimal delay [9,[13], [14], [15]]. In our cohort, the average image generation time per sample was approximately 8.2 min (acquisition of 4–5 images to cover a ∼1-cm segment of surgical margin). In comparison, cryo-HE processing typically takes 20–30 min for all margins, including tissue transport, freezing, sectioning, staining, and review. This substantial time advantage highlights the potential of NYU-AI to streamline intraoperative decision-making and reduce operative delays.

The portable nature of the NIO laser imaging system and automated analysis support its seamless integration into surgical workflows. Our study suggests that trained urologists may be able to interpret NYU-AI overlays in real time with reasonable reliability. Nevertheless, final surgical decisions based on histopathology remain the responsibility of board-certified pathologists. The goal of NYU-AI is not to replace but to assist pathologists by facilitating faster and more flexible intraoperative evaluations. This capability may prove particularly valuable during off-hours or in resource-limited settings where rapid pathology input is unavailable. In this context, the AI-assisted interface functions as a complementary triage and communication tool within the multidisciplinary surgical team.

Previous studies demonstrated the potential of Raman-based and SRH imaging for prostate cancer diagnosis [[12], [13], [14], [15]]. The current validation adds to this body of work by confirming that an NYU-AI model developed for one context can be safely and effectively translated to another. Future directions include comparative analysis between AI-only, surgeon-only, and pathologist-augmented interpretation strategies, and integration into robotic workflows [15].

Growing interest in intraoperative margin assessment has been reinforced by recent high-level evidence demonstrating its clinical value. In particular, it has been shown that intraoperative frozen-section analysis during nerve-sparing prostatectomy improves functional outcomes without compromising oncologic safety [16]. However, widespread implementation of such strategies may be limited by the substantial time and pathology resources required for frozen section workflows. In this context, SRH imaging with AI support could serve as a digital alternative or adjunct and allow rapid intraoperative assessment of surgical margins with a lower logistical burden. Integration of NYU-AI into structured intraoperative strategies, such as the NeuroSAFE approach, may help in retaining the functional benefits of nerve-sparing surgery while streamlining decision-making and minimizing operative delays. Alongside SRH, other emerging platforms such as fluorescence confocal microscopy (FCM) have also demonstrated promising results. The LaserSAFE technique, an FCM-based system evaluated within the NeuroSAFE workflow, has shown high sensitivity and interobserver agreement for PSM detection during RARP [17]. These findings highlight a broader trend towards digital intraoperative pathology, whereby various optical modalities could be tailored to institutional needs and workflow constraints. In this broader landscape, NYU-AI offers distinct advantages such as portability, speed, and seamless AI integration, and may complement or even substitute for existing imaging techniques in future comparative trials. Future analyses may also incorporate quantitative margin distances to enhance risk stratification and surgical planning.

This first evaluation of the study has several limitations. First, the sample size was small (*n* = 18) owing to the preliminary nature of the study, which limits statistical power and generalizability. Second, the AI model used was originally trained on SRH images from surgical beds and needle biopsies, rather than on prostatectomy specimens, which may influence diagnostic performance in this specific anatomic context. Third, the study was conducted at a single academic center, and all NYU-AI images were interpreted by a single blinded urologist. Therefore, interobserver agreement was not assessed, and external validation across multiple institutions and clinical readers is required to confirm reproducibility. Importantly, this report represents the preliminary and AI-focused analysis phase of the ongoing ROBOSPEC study, which is designed to further investigate the clinical utility of SRH in a broader patient population and across diverse procedural settings. Fourth, as the goal is to use NYU-AI as an intraoperative diagnostic adjunct, cryo-HE, which is the current standard for intraoperative margin assessment, was chosen as the appropriate comparator in this preliminary phase. Although FFPE histopathology was performed as part of routine care, direct comparisons with NYU-AI were not conducted in this analysis and should be addressed in future validation studies. Lastly, evaluation of extracapsular extension (ECE) was not within the scope of this preliminary analysis, and neither SRH nor cryo-HE was systematically used for ECE assessment in this cohort. Given the clinical significance of ECE in surgical planning and prognosis, this represents an important limitation. Future phases of the ROBOSPEC study may explore the potential of NYU-AI to contribute to intraoperative detection of ECE, particularly in high-risk cases.

## 5 Conclusions

This study demonstrates that an NYU-AI model originally developed for surgical bed and needle biopsy analysis retains diagnostic accuracy when applied to excised prostate margins. This validation confirms the robustness and clinical versatility of NYU-AI systems. Unlike traditional frozen-section analysis, NYU-AI allows rapid, label-free imaging with real-time interpretation and offers a scalable solution for intraoperative margin assessment during RARP. The high NPV supports its potential to minimize unnecessary resection while maintaining oncological safety. These promising results justify further prospective validation in larger, multicenter cohorts and open the door to seamless integration of AI-assisted pathology into surgical workflows.

  ***Author contributions***: Nikolaos Liakos had full access to all the data in the study and takes responsibility for the integrity of the data and the accuracy of the data analysis.

  *Study concept and design*: Özkan, Schröder, Beck, Werner, Gratzke, Straehle, Liakos.

*Acquisition of data*: Özkan, Schröder, Beck, Werner, Straehle.

*Analysis and interpretation of data*: Özkan, Liakos.

*Drafting of the manuscript*: Özkan.

*Critical revision of the manuscript for important intellectual content*: Özkan, Schröder, Bronsert, Franz, Glienke, Sigle, Taneja, Beck, Werner, Gratzke, Straehle, Mannas, Liakos.

*Statistical analysis*: Özkan, Sigle, Straehle.

*Obtaining funding*: None.

*Administrative, technical, or material support*: Özkan, Schröder, Bronsert, Taneja, Beck, Werner, Mannas.

*Supervision*: Liakos, Gratzke.

*Other* (*validation*): Özkan, Schröder, Franz, Glienke, Sigle, Liakos.

  ***Financial disclosures:*** Nikolaos Liakos certifies that all conflicts of interest, including specific financial interests and relationships and affiliations relevant to the subject matter or materials discussed in the manuscript (eg, employment/affiliation, grants or funding, consultancies, honoraria, stock ownership or options, expert testimony, royalties, or patents filed, received, or pending), are the following: None.

  ***Funding/Support and role of the sponsor*:** None.

  ***Ethics statement***: The study was conducted in accordance with the ethical standards of the institutional and national research committees and with the Declaration of Helsinki and its later amendments. The study was registered in the Freiburg Study Registry under registration number 24-1266-S1after receiving ethics approval from the ethics committee of the University of Freiburg.

  ***Data sharing statement***: All data are available in full from the corresponding author.
